# Thirteen cases of pulmonary dirofilariasis in a single institution in Okinawa Island

**DOI:** 10.1007/s00428-019-02614-9

**Published:** 2019-06-28

**Authors:** Eriko Atsumi, Hirofumi Matsumoto, Naohiro Taira, Tomofumi Yohena, Hidenori Kawasaki, Tsutomu Kawabata, Naoki Yoshimi

**Affiliations:** 1 Division of Pathology, National Hospital Organization, Okinawa National Hospital, 3-20-14, Ganeko, Ginowan, Okinawa 901-2214 Japan; 2 Department of Pathology and Oncology, Graduate School of Medicine, University of the Ryukyus, 207, Uehara, Nishihara, Okinawa 903-0215 Japan; 3 Division of Surgery, National Hospital Organization, Okinawa National Hospital, 3-20-14, Ganeko, Ginowan, Okinawa 901-2214 Japan

**Keywords:** *Dirofilaria immitis*, Epidemiology, Histopathology, Necrotic nodule, Pulmonary dirofilariasis

## Abstract

Pulmonary dirofilariasis is an infection caused by *Dirofilaria immitis*, which is an endemic parasite in Japan. We experienced 13 surgical cases of pulmonary dirofilariasis in our hospital. Of the 13 patients, 61.5% were men. The responsible lesions were located in the right lung in all cases, and 76.9% of them were in the lower lobe. Histologically, 12 cases showed necrotic nodules with peripheral granuloma with worms inside the pulmonary artery. One case did not show a necrotic nodule but showed only thickening and hyalinization of the pulmonary artery wall with a degenerated worm inside. Eosinophils were found histologically in all cases. Thirteen cases of dirofilariasis in one institution seem to be the largest number in Japan, based on previous reports. One reason for this increased prevalence may be the hot and humid climate of our prefecture considering the ecology of the mosquito as a vector. Elastic staining and eosinophils in peripheral granulomatous areas can contribute to the diagnosis when the worms are degenerated.

## Introduction

Pulmonary dirofilariasis is a nematode infection caused by *Dirofilaria immitis* (*D. immitis*), which is an endemic parasite in Japan among canines and felines. It is transmitted to humans by bites from mosquitos that have sucked the blood of infected animals. Since the human is not a final host, the larvae die in the heart before maturing into adult worms and are washed out into the lungs, where they form a nidus of the thrombus in the pulmonary artery [1]. Most cases are asymptomatic and usually are noted as a lung nodule on chest X-ray or computed tomography (CT) by chance. We experienced 13 cases of pulmonary dirofilariasis that underwent surgery between 2000 and January 2018.

## Materials and methods

All 13 resected dirofilariasis cases diagnosed between 2000 and January 2018 were included in this study. Serum antibodies for dirofilariasis were examined using a multiple dot enzyme-linked immunosorbent assay [2], and the evaluation criteria are as follows: 3, positive; 2, weakly positive; 1, equivocal, and 0: negative. Hematoxylin and eosin (H&E) and elastic (either elastic van Gieson stain or elastic H&E) staining were performed in all cases. The worms were identified by their characteristic thick cuticle [3]. Eosinophils were counted based on the method and criteria of Araya et al. [4]. Cell counting was performed in 3 representative fields at × 400 magnification on H&E-stained slides, and the average counts were classified into the following 3 categories: category 0: 0–2 eosinophils in 1 high-power field (HPF); category 1: 3–50 eosinophils in 1 HPF; and category 2: > 50 eosinophils in 1 HPF. All cases were summarized using clinical, radiological, and histopathological characteristics. Cases 1, 2, and 3 were reported previously by Oshiro Y et al. [5].

## Results

Between 2000 and January 2018, 3510 patients underwent thoracic surgery in our hospital for lung diseases, mediastinal diseases, and pleural diseases. Of the lung disease patients, 139 were diagnosed with benign nodular lesions, including 13 dirofilariasis cases, which represented 9.4% of the benign nodular lesions.

The clinical and radiologic characteristics are shown in Table 1. Of the 13 patients, 8 cases were men (61.5%), and the average age was 63.2 years. In 12 cases (92.3%), the pulmonary nodule was noted by chest imaging prior to surgery; of these cases, 7 were identified by health check, 4 by chest imaging during follow-up or workup for other diseases (1 colon cancer, 1 chronic obstructive pulmonary disease with asthma, 1 meningioma, and 1 biliary colic), and 1 was incidentally found by chest imaging taken for chest and back pain unassociated with dirofilariasis. Case 13 underwent lobectomy for lung adenocarcinoma, and dirofilariasis was incidentally found histologically in the noncancerous part of the resected lung. Case 2 showed 2 nodules in 1 patient, both of which were diagnosed as dirofilariasis. Peripheral blood eosinophils were examined in all cases, and only 3 cases showed eosinophilia (7.4%, 11.9%, and 21.3%). The average percentage of peripheral eosinophils was 4.9%. Serum antibodies for dirofilariasis were examined in 9 cases, and the results were as follows: 1 positive, 1 weakly positive, 5 equivocal, and 2 negative. In all cases, the responsible lesions were found in the right lung, and in most cases were found in the lower lobe (76.9% in the lower lobe and 23.1% in the upper lobe).

**Table 1:** Clinical characteristics of the 13 cases

Case	Age	Sex	Reason for visiting the hospital	Site	Eosinophils % (count)	Size (mm)	Antibody
1	65	M	Health check	RLL	2.9 (179.22)	19	1
2	77	M	Follow-up of meningioma	RLL (S6, S9)	1.5 (107.7)	12	1
3	68	M	Workup of biliary colic	RLL	1.1 (115.06)	20	0
4	37	F	Health check	RLL	0.4 (21.84)	20	2
5	75	F	Health check	RLL	21.3 (1399.41)	17	3
6	75	F	Health check	RLL	4.9 (328.79)	8	NE
7	70	M	Health check	RUL	7.4 (410.7)	24	1
8	46	F	Left chest back pain	RLL	1.1 (70.29)	25	0
9	59	M	Health check	RLL	1.9 (80.18)	20	1
10	72	M	Follow-up of colon cancer	RUL	4.9 (268.03)	10	NE
11	53	M	Health check	RUL	2.3 (127.65)	14	NE
12	60	M	Follow-up of asthma and COPD	RLL	11.9 (1062.67)	17	1
13	64	F	Incidentally found in resected lung	RLL	1.4 (68.46)	–	NE

*RLL*, right lower lobe; *RUL*, right upper lobe; *NE*, not examined

Serum antibody criteria: 3, positive; 2, weakly positive; 1, equivocal; 0, negative

The nodule size of case 2 is the largest diameter of the 2 nodules

## Histopathological findings

**Fig. 1:**
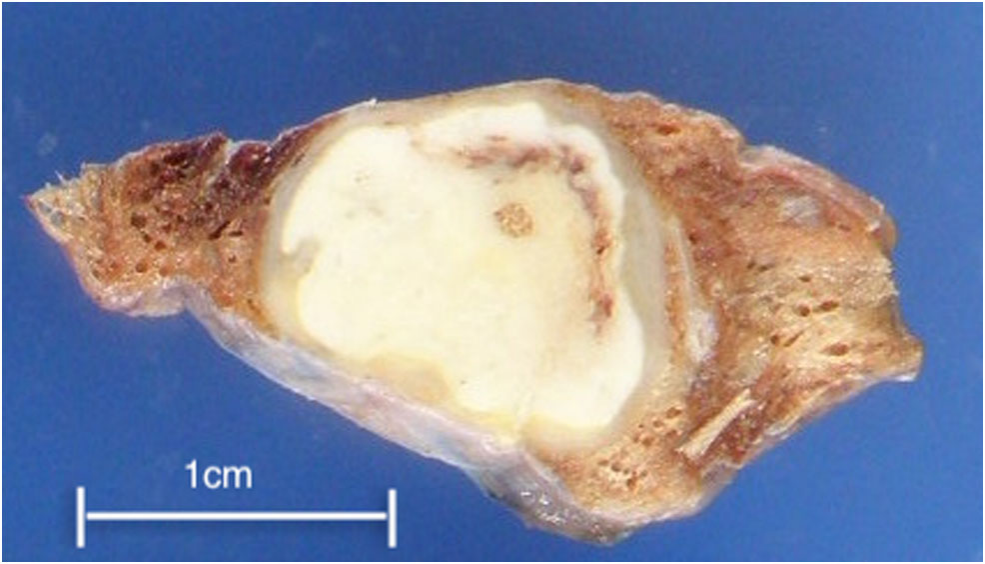
Macroscopically, all but one case showed a well-circumscribed yellowish-white nodule

**Fig. 2:**
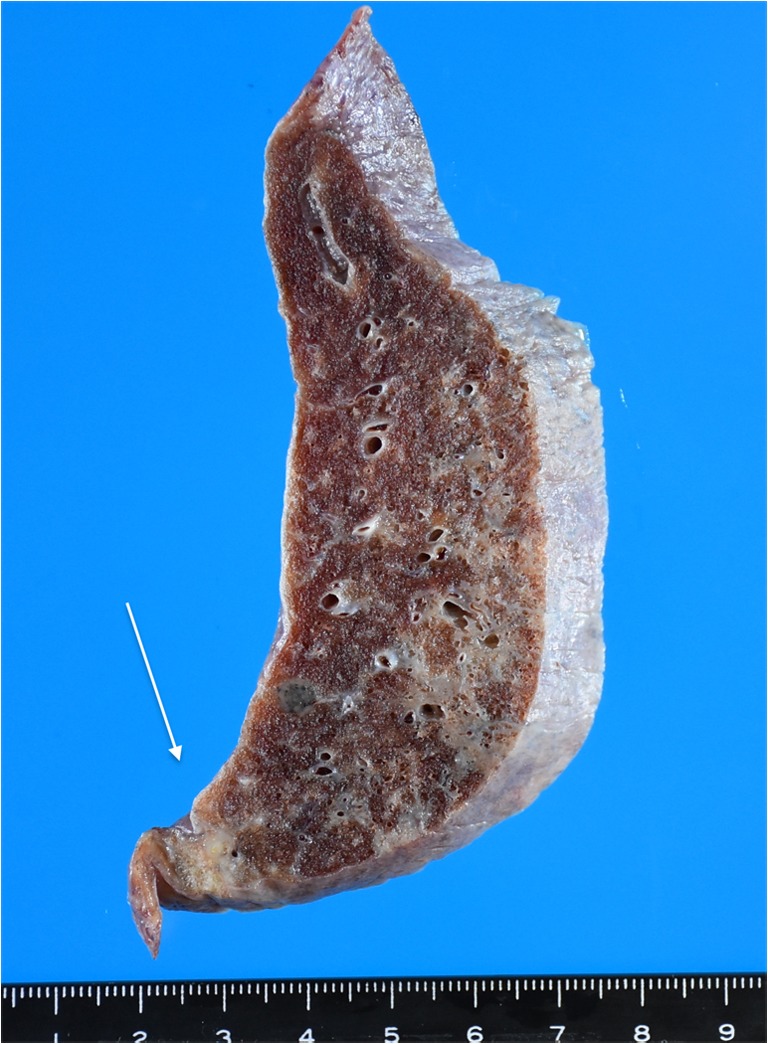
Macroscopically, case 13 showed a nodular to irregularly shaped lesion (arrow)

**Fig. 3:**
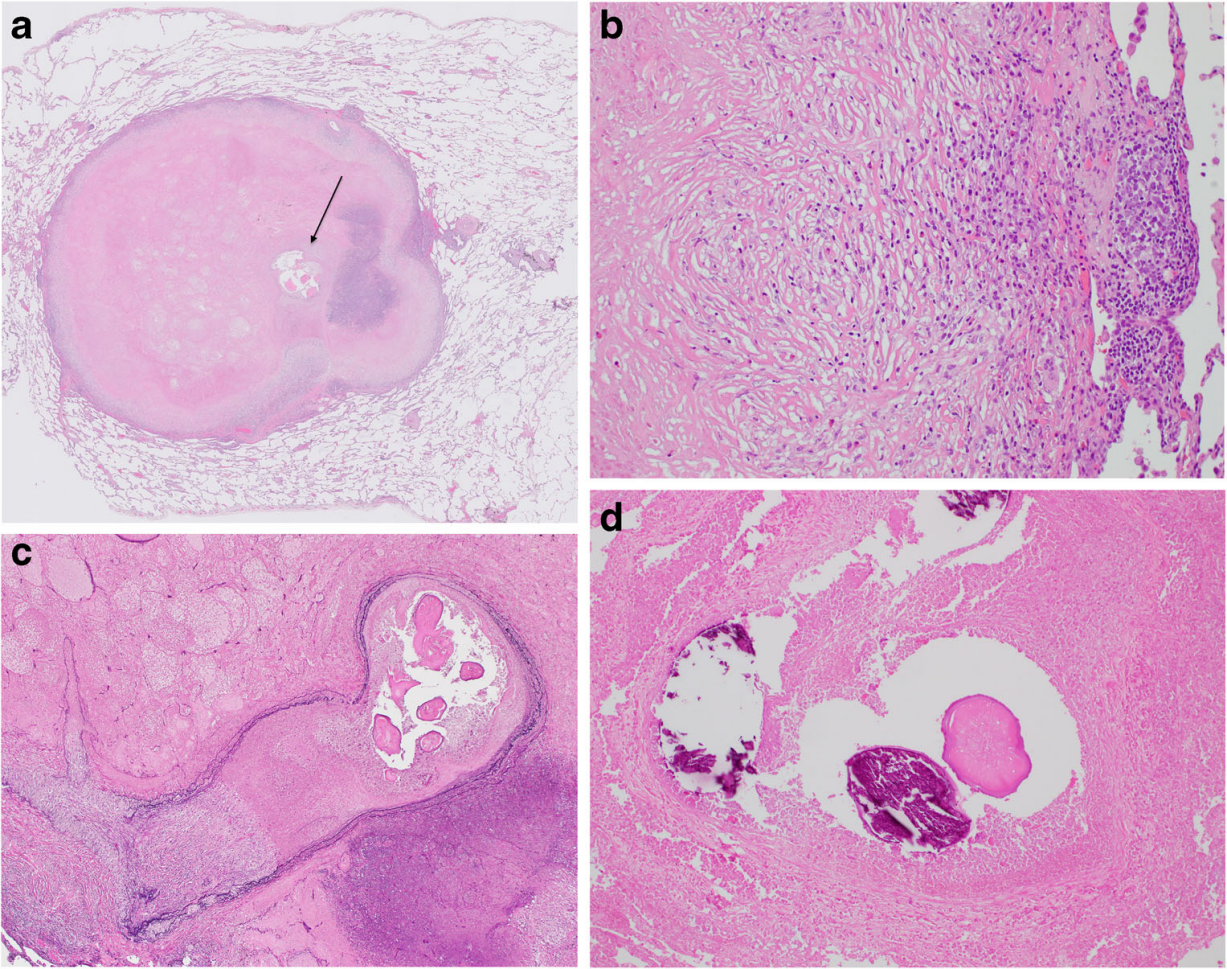
**a** Scanning magnification of the lesion showed a necrotic pink nodule with a blue rim composed of a granuloma and inflammatory cells. The worms were found in the cavity which was pulmonary artery (arrow) in H&E staining. **b** The rim of the nodule was composed of a granuloma and inflammatory cells, such as lymphocytes and eosinophils. Lymphoid follicle was also seen (H&E staining, × 200). **c** The worms were located inside the pulmonary artery, as shown by elastic staining (× 40). **d** Some of the worms showed degenerative changes with calcification (H&E staining, × 100)

**Fig. 4:**
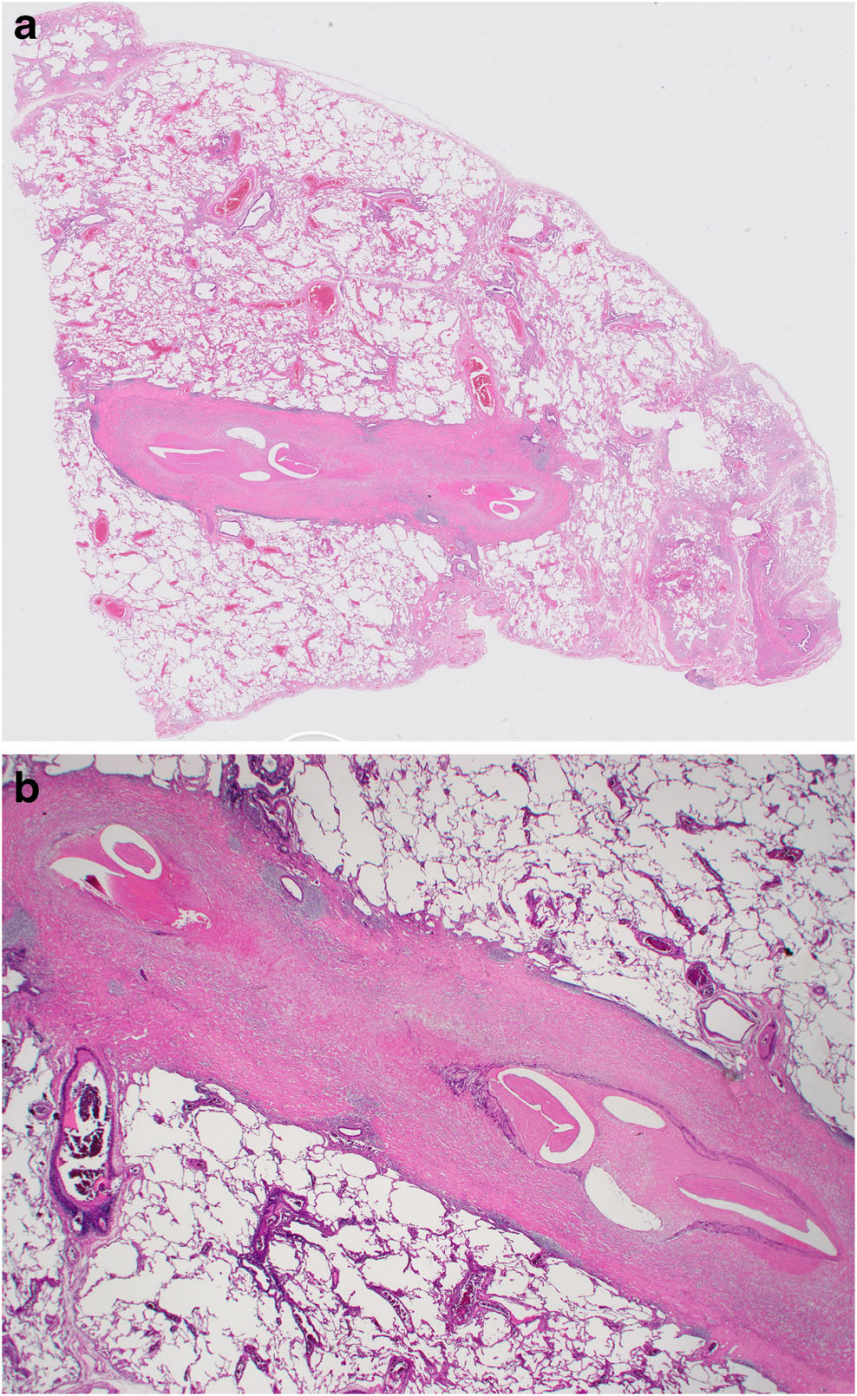
**a** Scanning magnification of case 13 showed a club-like structure instead of a typical necrotic nodule (H&E staining). **b** The club-like structure was revealed as a thickened and hyalinized vascular wall by elastic staining (× 12.5)

Macroscopically, all but one case showed a well-circumscribed nodule (Fig. 1). The average size was 17.2 mm, with a range from 8 to 25 mm. Case 13 did not show a clear nodular lesion but instead appeared as a small nodular to irregularly shaped lesion (Fig. 2). Microscopically, 12 cases showed necrotic nodules with peripheral granuloma consisted of lymphocytes, eosinophils, plasma cells, neutrophils, and occasionally giant cells (Fig. 3a, b). Some lymphoid follicles were seen in the granulomatous area of all cases. Worms were found in the thrombosed pulmonary artery in the necrotic area, as revealed by elastic staining (Fig. 3c). The average cross-sectional diameter of the worms was about 225 μm. Elastic staining also revealed preserved underlying structure in the necrotic area, which confirmed coagulative necrosis. The elastic layer of the pulmonary artery was partly destroyed in various proportions in 11 of the 13 cases (84.6%). In some cases, extravasated worms were seen outside the pulmonary artery. Many worms showed degenerative changes, and 1 case showed calcification (Fig. 3d). Case 13 did not show a necrotic nodule (Fig. 4a) but instead showed extensive thickening and hyalinization of the pulmonary artery wall, and the degenerated worms were found inside the pulmonary artery (Fig. 4b). The elastic layer of the pulmonary artery was partly destroyed, but no extravasation of the worm was seen (Fig. 4b).

Eosinophils were found in peripheral granulomas in all cases as follows: category 1: 8 cases (61.5%) and category 2: 5 cases (38.5%).

## Discussion

We experienced 13 cases of dirofilariasis in a single institution over an approximately 18-year period. Miyoshi et al. [6] reviewed the literature and demonstrated that 24 dirofilariasis cases were reported in Japan from 1998 to 2004. To the best of our knowledge, 13 cases in 18 years from a single institution is the largest number in Japan, based on previous reports [6]. We inferred that the frequent occurrence was related to the climate of Okinawa, which is located in a subtropical region and is the southernmost region of Japan. The life cycle of *D. immitis* is heavily dependent on climate. Genchi et al. reported that *D. immitis* development would not proceed in conditions with a temperature below 14 °C [7]. Fortin and Slocombe demonstrated that the duration needed for the development of *D. immitis* microfilariae to infective larvae inside mosquitoes was dependent on temperature (i.e., 8–9 days at 30 °C, 10–14 days at 26 °C, 17 days at 22 °C, and 29 days at 18 °C) [8]. Because most infected mosquitoes in the wild cannot survive for more than 30 days [9], the prevalence rate of *D. immitis* would be low in areas with a cold climate.

Since our institution stands in the mid-south area of Okinawa’s main island, which is in a subtropical area as mentioned above, it is hot and humid throughout most of the year. Mosquitos species such as *Culex quinquefasciatus* and *Aedes albopictus*, which are important vectors of natural *D. immitis* infections in Japan, propagate throughout the year on Okinawa Island [10]. As a consequence, the seroprevalence of *D. immitis* infection among dogs is expected to be higher in Okinawa than in the other region of Japan. For example, the seroprevalence of *D. immitis* infection among shelter dogs was 36.1% in Okinawa Island between 1991 and 1992 [10], on the other hand, it was 23% between 2009 and 2011 in Tokyo [11]. The average temperatures of Okinawa and Tokyo during that period were 23.2 °C and 16.7 °C, respectively [12].

Furthermore, the daily variation of the temperature is milder in Okinawa than in Tokyo [12], which allows mosquitos’ high activity all day long. As reported previously, it is difficult for *D. immitis* to mature inside the mosquitos and become infectious larvae under 18 °C [8, 9]. Therefore, it means that the climate in Okinawa allows mosquitos to be vectors for *D. immitis* almost throughout the year, resulting in the increased seroprevalence of *D. immitis* among dogs, thus leading to the high incidence of human dirofilariasis in Okinawa.

Another potential explanation is that knowledge about parasitological diseases in dogs is not sufficient. According to data from the Ministry of Health, Labor and Welfare, immunization coverage against canine rabies in Okinawa was lower than the average rate in Japan (50.1% vs. 71.4%) in 2016 [13]. Although we could not find the coverage rate for dirofilariasis, we could infer that the coverage rate for *D. immitis* would also be low in Okinawa.

Interestingly, all 13 of our cases were found in the right lung, and about 80% of them in the lower lobe. Flieder and Moran reported 41 dirofilariasis lesions in 39 patients and found that 76% were in the right lung, especially in the right lower lobe (46% of all cases) [14]. Asimacopoulos et al. reported that 6 of their 10 cases were in the right lung; of those, 5 cases were in the lower lobe [15]. Milanez de Campos reported lower lobe predominance in their 24 cases, especially right lower lobe [16]. Flieder inferred the reason for the right lower lobe predominance to be a larger surface area and increased blood flow [14]. In addition, an anatomical difference exists in that the right pulmonary artery runs straight through the anterior part of the right main bronchus, which is in contrast to the left pulmonary artery, which crosses over the left main bronchus. These reasons must explain why embolization by worms tends to occur on the right side, especially in the lower lobe.

Pathologically, approximately 90% of the cases showed a typical necrotic nodule surrounded by granulomatous inflammation. However, our case 13 showed only thickening and hyalinization of the pulmonary artery wall instead of the typical histological feature. Degenerative changes in the worm suggested that this lesion was old, and therefore, the creation of a necrotic nodule afterward was unlikely in this case. Araya et al. hypothesized that 2 mechanisms contributed to the creation of the spherical necrosis of dirofilariasis. The first reason is Th2-type cytokine production by inflammatory cells, which are recruited in response to worm antigens, and the second reason is an altered blood supply [4]. In our case 13, only small numbers of eosinophils were found in and around the hyalinized pulmonary artery wall, and an inflammatory response including lymphocytes was not prominent. Thus, one explanation for the discrepancy of this case was that the inflammatory response was not sufficient to produce cytokines and form a necrotic nodule. The elastic layer was at least partly destroyed in 84.6% of the cases, and some worms were extravasated; the worm antigens may be stronger if the worm extravasates from the pulmonary artery to the adjacent lung tissue and thus stimulate an inflammatory response. In case 13, the elastic layer was partly destroyed, but the extravasation of the worm was not observed. In addition, case 13 patient had 4-cm untreated solid adenocarcinoma with pleural and vascular invasion. Thus, one possibility is that this patient’s immunity was not completely innate, and impaired immunity altered the histological findings of this case. However, in some cases, a necrotic nodule formed despite a rather poor inflammatory response. Though there are some reports of dirofilariasis cases which showed pleural effusion only with no nodules on chest imaging [17, 18], these are not resected cases. To the best of our knowledge, no previous study has reported a resected pulmonary dirofilariasis case that has not formed a necrotic nodule. Further investigation with more cases is needed to reveal the reason why this case did not form a necrotic nodule as other cases. In addition, eosinophils were found in the peripheral granulomatous areas of all cases, with mild to moderate cases accounting for approximately 60% of the total, which was in agreement with a previous report [4]. Eosinophils found histologically will aid with the diagnosis of a resected specimen, especially when the worms are already degenerated and visualizing their characteristic structure is difficult. Peripheral eosinophilia was observed only in limited cases, which was similar to a prior report [6, 14, 16], and did not seem to be useful for the diagnosis. Serum antibodies for dirofilariasis also did not show high sensitivity, since only 2 cases were positive, including 1 weakly positive. Thus, these results indicate that diagnosing dirofilariasis using laboratory tests before an operation is difficult, as mentioned in previous reports [14, 16].

## Conclusion

We experienced 13 resected dirofilariasis cases. The incidence rate seemed higher than most of the Japanese area, and we infer that the subtropical climate is one reason. Though Japanese land is small, we have various climates from subarctic to subtropical, and it is essential to recognize the specific diseases in each area. Since dirofilariasis typically present as asymptomatic nodules, it is easily mistaken as the neoplastic disease by chest imaging and is important to recognize the dirofilariasis as the differential diagnosis of pulmonary nodules. Diagnosis can be made by the resected specimen, but the time between infection and diagnosis would be long, which cause the worms degenerative and destructive changes and increases the risk of misdiagnosis. The worms’ fragments should be looked for carefully in cases with a necrotic nodule with a peripheral granuloma. Elastic staining can help indicate the pulmonary artery structure and confirm the presence of worms inside it and eosinophils in peripheral granulomatous area would be also helpful for the diagnosis.
